# Microbiota entrapped in recently-formed ice: Paradana Ice Cave, Slovenia

**DOI:** 10.1038/s41598-021-81528-6

**Published:** 2021-01-21

**Authors:** Janez Mulec, Andreea Oarga-Mulec, Ladislav Holko, Lejla Pašić, Andreja Nataša Kopitar, Tina Eleršek, Andrej Mihevc

**Affiliations:** 1 Karst Research Institute, Research Centre of the Slovenian Academy of Sciences and Arts, Titov trg 2, 6230 Postojna, Slovenia; 2 UNESCO Chair on Karst Education, University of Nova Gorica, Glavni trg 8, 5271 Vipava, Slovenia; 3 School of Environmental Sciences, University of Nova Gorica, Glavni trg 8, 5271 Vipava, Slovenia; 4 Institute of Hydrology of the Slovak Academy of Sciences, Dúbravská cesta 9, 84104 Bratislava, Slovak Republic; 5 School of Medicine, University Sarajevo School of Science and Technology, Hrasnička cesta 3a, 71000 Sarajevo, Bosnia and Herzegovina; 6 Faculty of Medicine, Institute of Microbiology and Immunology, University of Ljubljana, Zaloška 4, 1000 Ljubljana, Slovenia; 7 Department of Genetic Toxicology and Cancer Biology, National Institute of Biology, Večna pot 111, 1000 Ljubljana, Slovenia

**Keywords:** Ecology, Microbiology, Ecology, Environmental sciences

## Abstract

Paradana is one of the biggest ice caves in Slovenia, with an estimated ice volume of 8,000 m^3^. Reflecting climatological conditions, the cave ice undergoes repeated freeze-thaw cycles and regular yearly deposition of fresh ice. Three distinct ice block samples, collected from the frozen lake in May 2016, were analysed to obtain data on ice physicochemical properties and the composition of associated microbiota. Isotopic composition of the ice samples (^18^O, ^2^H) and a local meteoric water line (LMWL) constructed for monthly precipitation at Postojna were used to estimate the isotopic composition of the water that formed the ice, which had high values of deuterium excess and low concentrations of chloride, sulphate and nitrate. The values of total organic carbon (1.93–3.95 mg/l) within the ice blocks fall within the range of those measured in karst streams. Total cell count in the ice was high and the proportion of cell viability increased along the depth gradient and ranged from 4.67 × 10^4^ to 1.52 × 10^5^ cells/ml and from 51.0 to 85.4%, respectively. Proteobacteria represented the core of the cave-ice microbiome (55.9–79.1%), and probably play an essential role in this ecosystem. Actinobacteria was the second most abundant phylum (12.0–31.4%), followed in abundance by Bacteroidetes (2.8–4.3%). Ice phylotypes recorded amounted to 442 genera, but only 43 genera had abundances greater than 0.5%. Most abundant were *Pseudomonas*, a well-known ice dweller, and *Lysobacter*, which previously was not reported in this context. Finally, two xanthophytes, *Chloridella glacialis* and *Ellipsoidion perminimum*, known from polar environments, were cultured from the ice. This indicates that the abundance and ecological role of phototrophs in such environments might be greater than previously deduced.

## Introduction

Cold ecosystems include deep oceans, polar regions, high mountains and subterranean caves with a large variety of aquatic and terrestrial ecosystems^1^. In recent years, cold habitats such as glaciers and ice sheets^2^, sea ice^3^, lake ice^4^ and permafrost^5^ have received most attention as regards microbiological studies, mainly because of their sensitivity to climate change. Also, there is a growing research interest related to frozen ecosystems as analogues of extraterrestrial habitats^6^. Even if the study of these habitats is supported strongly by the rapid development of new technologies (which shed more light on the gene presence, functional gene potential, gene expression, in situ identification of active microorganisms and biotechnological potential) more fundamental ecological research is needed urgently. In this respect there is a lack of information on the dynamics and functions of microbial communities, their management and predictive models, interactions between micro- and macroorganisms in cold habitats, linkage of biodiversity, climate, and function and finally an integrated view encompassing micro- and macroorganisms and the physical environment^1^.

Ice deposits in caves have been poorly studied as microbial habitats, despite providing important ecological information on microbiota living close to what are normally considered boundary living conditions for some species. The few available studies on the cave-ice microbiome all indicate its high diversity^7–10^. Besides, ice strata of different ages (Recent down to 13,000 BP) from Scărişoara Ice Cave in Romania revealed microbiota as an integral part of the ice in terms of diversity, quantity, and activity^11–14^. Low temperatures in this cryosphere slow down or stop some metabolic and environmental processes and preserve the habitats as “time capsules”, sources of nutrients and assemblages of organisms. Besides microbiota, ice deposits in caves represent an invaluable archive of paleo-environmental data^15^. Data from Scărişoara Ice Cave served to construct a model of Holocene winter climate variability in Central and Eastern Europe^16^. Reconstruction of past climates is not the only application of ice-cave data. Tritium and ^14^C data from ice caves allow discrimination of the period of ice formation (before or after the 1960s) and age of ice^17–19^. Stable isotopes of oxygen and hydrogen and chemical composition provide information on the origin of the ice, e.g. local precipitation, karst and/or surface water^17, 18, 20^.

Because of the accelerated trend of ice-melt worldwide^21^, investigation of ice caves is now crucial as a step towards collecting and preserving essential climatic and geological data, as well as information about its associated life and related genetic pool. Particularly threatened with decay and disappearance is cave ice, which represents only a fraction of the terrestrial cryosphere. Its impending disappearance is especially evident in karst caves at lower altitudes in temperate zones.

The study presents details of the physicochemical characteristics of cave ice, and focuses on the structure of the associated microbiome in ice from the largest ice cave in Slovenia, as a potential basis for future research and comparison with other ice caves. Here, the detail of cave-ice deposition is largely impacted by snow levels, the presence of external organic debris, the influence of cave percolation water, and the effects of strong air circulation. Such extreme environmental conditions could direct the compartmentalization of microbial habitats.

## Results and discussion

### Ice environment

Physicochemical analyses of individual ice blocks were conducted to observe eventual differences that could be attributed to spatially related gradual freezing–melting and fresh ice deposition, and to characterize the habitat that enables long-term survival of ice microbiota. All ice samples contained low concentrations of salts, indicating that they originated from recent clean snow. Concentrations of anions in the upper layers, Ice-1 and Ice-2, were similar. However, the bottom layer Ice-3 had distinctly higher electrical conductivity (EC), hardness and alkalinity, less nitrate, and more sulphate. This could indicate that this ice stratum includes a higher proportion of percolation water, which contains more ions than rain and snow as shown by the differences between the percolation water from the cave Planinska jama (that was used for preparing growth media) and the ice, as shown in Table 1. Total organic carbon (TOC) concentrations in the ice were in a range typical of karst streams^22^, and above the minimum values reported for surface streams, i.e. 0.1–36.6 mg/l^23^, indicating a significant input of organic matter for the underground ecosystem. TOC indicates an available in situ source of carbon for the ice microbiome. Nitrogen expressed as nitrate did not exhibit high values in ice samples (Table 1). In this respect, a parallel can be drawn with karst sediments, where microbes are commonly limited more by carbon and phosphorus than by nitrogen^24^.

**Table 1:** Characteristics of ice samples from Paradana.

Parameter	Ice-1	Ice-2	Ice-3
pH	8.21	8.62	8.46
EC (μS/cm)	31	35	72
Hardness (mg/l CaCO_3_)	16.0	18.0	40.5
Alkalinity (mg/l CaCO_3_)	16.5	16.5	38.0
Cl (mg/l)	1.0	0.5	0.6
NO_3_ (mg/l)	1.1	1.3	0.2
SO_4_ (mg/l)	2.2	1.6	6.7
o-PO_4_ (mg/l)	0.0	0.0	0.0
TOC (mg/l)	1.93	3.95	3.19
δ^18^O (‰)	− 6.82	− 6.70	− 10.02
δ^2^H (‰)	− 41.6	− 40.3	− 62.7
Deuterium excess (‰)	13.0	13.3	17.5
ATP (RLU)	43	31	149
Total cell counts (cells/ ml)	4.67 × 10^4^	5.45 × 10^4^	15.15 × 10^4^
Cells viability (%)	67.4	51.0	85.4
r-strategists (%)	92.4	44.5	54.4

Besides EC and temperature, pH and dissolved oxygen are additionaly two influential parametres that can affect the abundance and taxonomic structure of microbial communities. pH was found to drive the shift in the community structure not only in habitats such as freshwater, marine sediments or soils but also in cold habitats as Antarctic soils^25^. In the current samples, the pH effect on the microbial community structure is less evident because all the values are rather similar (Table 1). Cave ice habitats with incoming waterflow are probably not oxygen depleted; on the contrary, for example in Antarctic lakes, glacial meltwater inflow is responsible for oxygen supersaturation^26^.

Isotopically, the Ice-3 stratum was significantly lighter than the stratum represented by Ice-1 and Ice-2 (Table 1). Correlation of δ^2^H and deuterium excess did not indicate any effect of kinetic fractionation during water freezing. Thus, intersection of the freezing-line determined by stable isotopes in samples Ice-1 to Ice-3 (δ^2^H = 6.48δ^18^O + 2.88) with the local meteoric-water line (LMWL) constructed for the precipitation station at Postojna (Supplementary Fig. S1) (δ^2^H = 7.95 δ^18^O + 12.13), provided the δ^18^O value − 6.3‰ for the original water before freezing. It represents relatively enriched water, but such a value is not uncommon in daily precipitation in Slovenia^27^. The ice lake in Paradana is presumably formed by the refreezing of water from melting snow accumulated during the winter months^20^, with some contribution of water dripping from the cave ceiling. November and December 2015 had only a few days with precipitation in Postojna (5 and 4, respectively). However, January and February 2016 had 12 and 20 days with precipitation and monthly totals were high, 152 mm and 312 mm, respectively. The air temperature data adjusted for the elevation difference between Postojna and the Trnovski gozd karst plateau (about 600 m) indicate that about one third of the precipitation in January and one half in February probably fell as snow. The rest was probably a mixture of solid and liquid precipitation, but heavy rains could have occurred as well (e.g. about 55.5 mm of precipitation was measured in Postojna on February 8–9, with mean daily air temperatures between 8 °C and 9 °C). Isotopic composition of precipitation varied significantly between and also during individual events. It is known that snow cover can preserve the isotopic composition of the original snowfalls for long periods^28^. However, individual snowfalls can mix at the entrance of the cave and the isotopic composition of snow accumulated in the cave can also be influenced by thaws caused by temporary increases of air temperature or rainfall. The isotopic composition of snowmelt water that eventually refreezes in the cave is therefore the result of many processes. Further research with better temporal and spatial resolution of samples and sampling of snowmelt water would be needed to improve knowledge on the dynamics and sources of ice formation. LMWLs known from the literature for other precipitation stations in Slovenia, i.e. Kozina, Portorož and Ljubljana that are given in Supplementary Fig. S1 provided δ^18^O values for the original water, which we consider too high (− 3.0‰ for LMWL from Portorož, − 3.8‰ for LMWL from Ljubljana and − 5,1‰ for LMWL from Kozina). Postojna is the closest precipitation station to the Paradana and the data on isotopic composition of precipitation cover the period of ice sampling (Supplementary Fig. S2). Therefore, the LMWL at Postojna could be the best representation of the isotopic composition of precipitation supplying water to the Paradana Ice Cave (after considering the elevation difference between the two sites, which is about 600 m).

When analysed in more detail, results obtained using the approach described above (to calculate the isotopic composition of the water that formed the sampled ice) also revealed the sensitivity of the constructed LMWL, the length of data series and extreme values. This is illustrated by records of isotopically very light precipitation in November and December 2015 (δ^18^O − 17.6‰ and − 14.2 δ^18^O, respectively). Although such isotopically light precipitation occurred in just two of the 27 months of the observation period, the two values changed the LMWL intercept significantly. However, because they did occur, they cannot be disregarded in the LMWL construction. Daily precipitation data indicate that in both cases monthly values were influenced dominantly by precipitation that fell during just one day (precipitation on those days represented almost the entire monthly precipitation). The LMWL intercept at Postojna without those two months would be 8.3, i.e. closely similar to values in Ljubljana and Kozina. Long-term data from Ljubljana show that the δ^18^O value of monthly precipitation was lower than − 16.0‰ (values around − 14.0‰ were quite abundant until 1986 and after 2004) in only 5 months in the years 1981–2010. Thus, precipitation with notable isotopically light values, as observed in Postojna between 21 and 23 November 2015 (92% of the precipitation fell on 22 November) appears to be rare in the study area. Nevertheless, it was observed, and it influenced the intercept of LMWL significantly.

It is worth noting that the δ^18^O values of Ice-1 and Ice-2 are higher than those reported for the Paradana Ice Cave by Carey et al.^20^. Deuterium excess is also significantly higher than the mean value reported for samples from different depths of ice by Carey et al.^20^. The difference in δ^18^O values could be related to different sampling sites. Carey et al.^20^ sampled the wall ice, whereas the samples collected during this study represent the frozen lake. Investigation of the difference in deuterium levels would be especially interesting. It could point at the input (either by overland flow from the cave entrance or by percolation from the vadose zone) of water from the autumn/winter months, with precipitation from the Eastern Mediterranean air masses having particularly high d-excess (up to 22‰). The Western Mediterranean air masses have d-excess of about 14‰, whereas air masses from the Atlantic have values of only about 10‰^29^. Late autumn to early winter precipitation in Slovenia (October to December) regularly exhibits high d-excess^27^. Unfortunately, the available data are insufficient to support analysis of the reason for high deuterium excess of the ice in detail. Study samples also display far lower concentrations of chloride, sulphate and nitrate than samples collected by Carey et al.^20^.

### Concentration of microbes in cave ice

The upper ice stratum represented by Ice-1 and Ice-2 had comparable microbial load expressed in total ATP concentration and total cell counts, whereas the Ice-3 block exhibited significantly higher values (Table 1). Interestingly, the total cell counts of microorganisms in the ice samples was similar (4.67 × 10^4^–15.15 × 10^4^) to that recorded in the Pivka River (SW Slovenia) at the ponor connecting to the karst underground, i.e. 4.29 × 10^4^–12.38 × 10^4, 30^. A large proportion (51.0–85.4%) of entrapped microbes in the ice were viable, showing that they were able to survive ice formation and melting, or even several freezing–melting cycles. A relatively high cell viability can be linked to the availability of compatible solutes, indicated by correspondingly high TOC (Table 1). Not only do sugars and polyols increase microbial resistance to freezing, they can also be used inside the cell as carbon and nitrogen sources^31^. Higher concentration of salts in Ice-3 block was accompanied by the highest total cell counts and percentage of viable cells (Table 1). In ice from Scărişoara Cave total cell counts varied from 0.84 × 10^3^ to 3.14 × 10^4^ cells/ml with corresponding viability from 28.2 to 84.9%, but no correlation was observed between the ice age (0–13,000 years BP) or depth (0–25 m) and the total number of cells or viability^14^.

The media types used in this study differed in their ability to stimulate the growth of colonies. In general, nutrient-poor media and low temperatures resulted in higher colony counts in all samples. This phenomenon has been reported previously in cave microbiology, but was not correlated with phylogenetic diversity of microbes obtained on the growth media^32^. After 28 days of incubation, samples grown on the oligotrophic medium with percolation water (PWA) and cultivated at 10 °C produced the highest colony counts (Table 2). In context this indicates that cave percolation water contains soluble compounds that are not present in tap water and which support the growth of cave-ice microorganisms. With respect to individual samples, the highest colony counts were found in the Ice-3 sample, i.e., 167.37‰ of all cell biomass, determined by flow cytometry (Table 2), and this sample also contained the highest concentration of nutrients (Table 1). Cultivable anaerobic bacteria and fungi were detected in all the ice samples (Table 2).

**Table 2:** Colony counts (colony-forming units—CFU/ml) and their proportion to total cell counts determined by flow cytometry (‰) at different cultivation conditions and media.

Temperature/ cultivation days	Ice-1 CFU (‰)	Ice-2 CFU (‰)	Ice-3 CFU (‰)	Ice-1 CFU (‰)	Ice-2 CFU (‰)	Ice-3 CFU (‰)
	NA			R2A		
37 °C/ 2	5 (0.11)	13 (0.23)	78 (0.52)	0 (0.00)	5 (0.09)	190 (1.25)
20 °C/ 14	85 (1.82)	150 (2.75)	5,025 (33.17)	**1,001 (21.43)**	366 (6.72)	10,900 (71.97)
10 °C/ 28	523 (11.19)	786 (14.42)	**10,567 (69.77)**	579 (12.40)	**850 (15.59)**	11,400 (75.27)
5 °C/ 28	**837 (17.92)**	**1,097 (20.12)**	5,810 (38.36)	740 (15.84)	829 (15.21)	**16,775 (110.76)**

	RIDA			RIDA-an		
37 °C/ 2	1 (0.02)	2 (0.04)	99 (0.65)	1 (0.02)	1 (0.02)	33 (0.21)
20 °C/ 14	45 (0.96)	**118 (2.16)**	55 (0.36)	4 (0.09)	4 (0.06)	230 (1.52)
10 °C/ 28	43 (0.92)	81 (1.49)	**253 (1.67)**	14 (0.30)	**35 (0.63)**	479 (3.16)
5 °C/ 28	**66 (1.41)**	96 (1.76)	230 (1.52)	**15 (0.31)**	26 (0.48)	**553 (3.65)**

	MEA			RIDA-Y&M-an		
20 °C/ 14	32 (0.68)	**80 (1.47)**	**736 (4.86)**	+	+	+
10 °C/ 28	**398 (8.53)**	39 (0.71)	331 (2.19)	+	+	+
5 °C/ 28	41 (0.88)	56 (1.03)	401 (2.65)	+	+	+

	TWA			PWA		
20 °C/ 14	**155 (3.32)**	145 (2.66)	**8,520 (56.25)**	103 (2.19)	326 (5.99)	12,038 (79.48)
10 °C/ 28	93 (1.98)	155 (2.84)	4,280 (28.26)	**1,242 (26.59)**	**1,957 (35.90)**	**25,350 (167.37)**
5 °C/ 28	110 (2.35)	**577 (10.58)**	5,983 (39.50)	295 (6.32)	810 (14.86)	12,525 (82.70)

In bold—highest value; *RIDA* RIDACOUNT total aerobic count; *RIDA-Y&M* RIDACOUNT yeast&mold rapid; *an* anaerobic, + presence, not countable.

Communities in the ice blocks differed in the representation of r-strategists, with their predominance in the Ice-1, and a big difference between Ice-1 and Ice-2, the two ice samples from the same stratum. Interestingly, a more-uniform community structure in terms of r-strategists was displayed in ice block Ice-2–Ice-3 (Table 1). R-strategists commonly dominate in uncrowded and unstable habitats where resources are temporarily abundant and available; with development of a community, r-strategists are gradually replaced by the slow-growing equilibrium K-strategists^33^.

Cultivation on different media showed that the ice contained metabolically diverse microorganisms, aerobic and anaerobic bacteria and fungi. Two species of yellow-green algae were also recovered in cultures from samples Ice-2 and Ice-3. The two cultivated species, *Chloridella glacialis* and *Ellipsoidion perminimum* (for identification see Supplementary Fig. S3), were also found in green ice from Antarctica^34^. It is known from results of previous studies that algae in ice can survive and even grow under such adverse conditions^34–36^. They can also be well adapted to low light and low water temperature; for example they can thrive under ice- and snow-cover where the available photosynthetic photon flux density is only around the photosynthetic compensation point^37^. In these terms, and particularly in ice caves with available light, algae and cyanobacteria should not be overlooked as an important part of the ice microbial community. Interestingly, in Himalayan-type glaciers, the algae-rich layers in ice cores were suggested as providing accurate boundary markers of annual layers^38^. It remains unclear whether algae can be applied similarly as boundary markers in cave ice. Their existence is already known from some caves, for example in Hungary in a small ice cave colonizing surfaces of the ice^39^, Romania in Scarişoara Ice Cave at the ice/water interface^40^ and in New Mexico, USA, in Zuni Ice Cave giving the distinctive greenish patina of the layered ice^35^.

### Bacterial community structure

Previous study of ice from the Paradana Ice Cave showed that it probably originates from local rainfall that reaches the cave as drip water after dissolving bedrock while percolating from the surface, and from snow that includes dust particles^20^*.* Thus, the largely impacted cave ice in Paradana has different sources, each bringing along a diverse and adaptable microbiota. 16S metagenomic analysis was conducted to describe the taxonomic composition of bacteria found in different ice blocks. Quality filtration of sequence readings gave a total number of 120,381 sequences in the three studied samples (Table 3). The number of operational taxonomic units (OTUs) varied from 185 in Ice-2 to 304 in Ice-1. This pattern was in alignment with values of alpha diversity parameters: extrapolated richness (Chao1), abundance-based coverage estimator (ACE) and Shannon index (Table 3). The rarefaction curves indicated that the diversity had been sampled sufficiently (Supplementary Fig. S4).

**Table 3:** Number of reads, OTUs, taxon richness and diversity indexes for cave ice samples.

Sample	Number of reads	Total OTUs	Estimated OTU richness	Diversity	
			Chao1	ACE	Shannon
Ice-1	89,041	304	326.14 ± 10.48	320.22 ± 8.73	3.49
Ice-2	85,886	185	192.5 ± 5.08	192.59 ± 6.85	1.56
Ice-3	99,705	286	353.26 ± 20.93	367.18 ± 10.05	1.69

**Figure 1:**
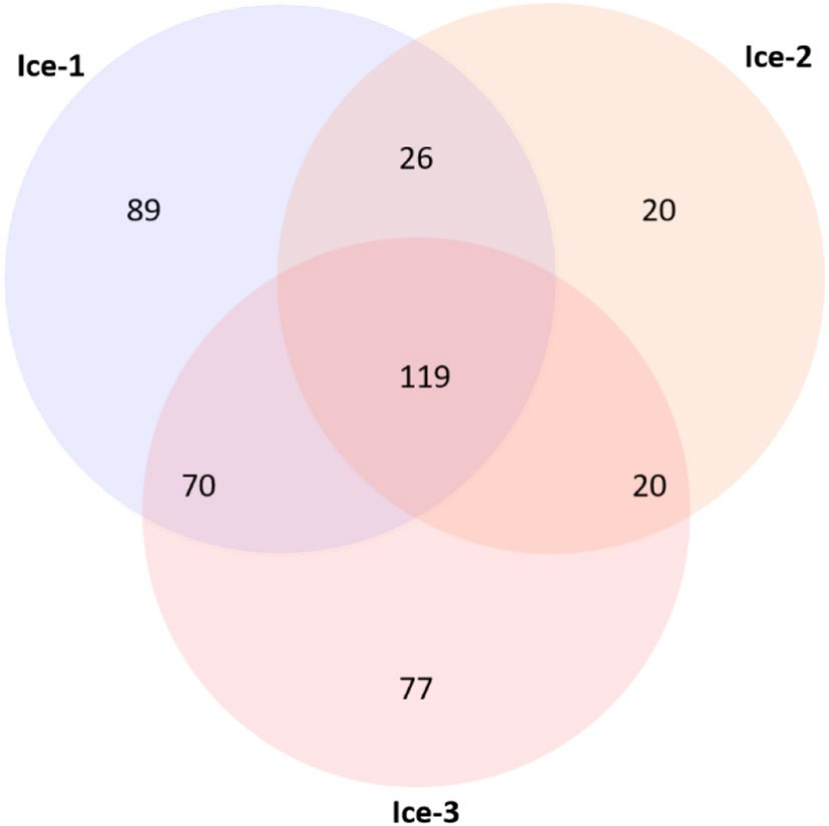
Prokaryotic OTU distribution in cave ice. The Venn diagram indicates the number of distinct and shared OTUs in ice samples Ice-1, Ice-2 and Ice-3.

A Venn diagram of the distribution of 441 distinct OTUs found in the three studied samples is presented in Fig. 1. Observations showed that 119 OTUs (28.3%) occurred in all three samples and can be interpreted as “a core microbiome”. Three of these OTUs dominated microbial communities in individual samples (relative abundance range 14.5–56.5%) and corresponded to the members of the genera *Pseudomonas*, *Lysobacter*, and *Sphingomonas*, as discussed below. These were followed in abundance by *Polaromonas*, *Flavobacterium*, *Rhodoferax*, *Nocardioides*, and *Pseudonocardia* (relative abundance range 3.3–6.9%). Another 35 OTUs had relative abundance above 0.5% and the remaining 76 OTUs had relative abundance below 0.5%. The unique OTUs probably contribute to the variability due to internal variations within the ice block caused by incoming snow or the freezing of percolation water. For example, samples Ice-2 and Ice-3 were cut from the same ice block in a vertical ice profile, but differed in their content of dark, particulate, organic inclusions.

**Figure 2:**
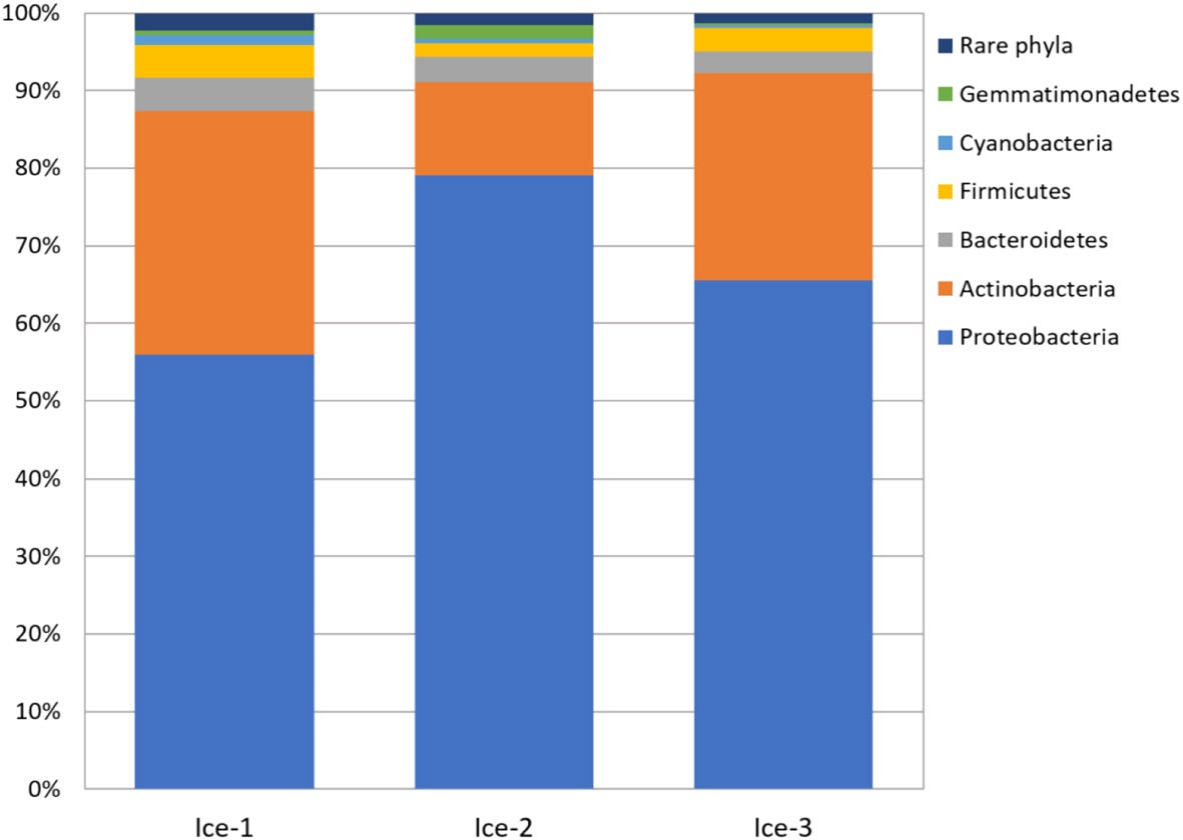
Relative abundance of phyla in the cave-ice samples. Phyla with relative abundance < 1.0% were classified together as “Rare phyla”.

Members of 29 bacterial phyla were detected in the cave ice microbiome (Fig. 2, Supplementary Fig. S5). All samples were dominated by Proteobacteria, with relative abundances of 79.1% in Ice-2, 65.5% in Ice-3 and 55.9% in Ice-1.

Proteobacteria commonly represent a dominant group in various cold habitats where their abundance is associated with increased nitrogen loads and copiotrophic growth conditions^41–43^. Indeed, Proteobacteria are commonly associated with water influx into the karst underground^44–46^. The prevalence of Proteobacteria in the ice samples coincided with the decreasing trend of TOC concentration in samples Ice-2, Ice-3 and Ice-1 (Table 1). This notion was further supported by a low relative abundance of members of the class Acidobacteria (0.1–0.3%, Supplementary Fig. S5), whose members are mostly oligotrophs^47^. Because Proteobacteria form the core of the cave-ice communities studied, it is likely that they play an essential role in the functioning of this recently formed ice ecosystem.

The second most abundant phylum in the studied samples was Actinobacteria, a group associated with cave sediment communities^48, 49^*.* Its relative abundance declined from 31.4% in sample Ice-1 to 26.7% in sample Ice-3 and 12.0% in sample Ice-2. This compares with the results from Scărişoara Cave, where the microbiome was dominated by Actinobacteria (38.5%) over Proteobacteria (33.5%), but Proteobacteria were assigned as the largest group of metabolically active microbes^14^.

In Paradana the relative abundance of Bacterioidetes phylotypes was comparably lower than that of the other two and was 4.3% in Ice-1, 3.2% in Ice-2, and 2.8% in Ice-3. Three other bacterial phyla represented > 1% of phylotypes in at least one sample and corresponded to Firmicutes, Cyanobacteria and Gemmatimonadetes. Phototrophic bacterial phylotypes belonging to Cyanobacteria were recovered from all three samples. They represented 1.3% of phylotypes in sample Ice-1, but only 0.6% and 0.3% in samples Ice-2 and Ice-3 respectively, from where algae, *C. glacialis* and *E. perminimum,* were obtained via cultivation.

Phyla whose relative abundance was less than 1% were grouped together and classified as “Rare phyla”. These phyla comprised 2.2%, 1.5% and 1.2% of Ice-1, Ice-2, and Ice-3, respectively. Their relative abundance is presented in Supplementary Fig. S5.

**Figure 3:**
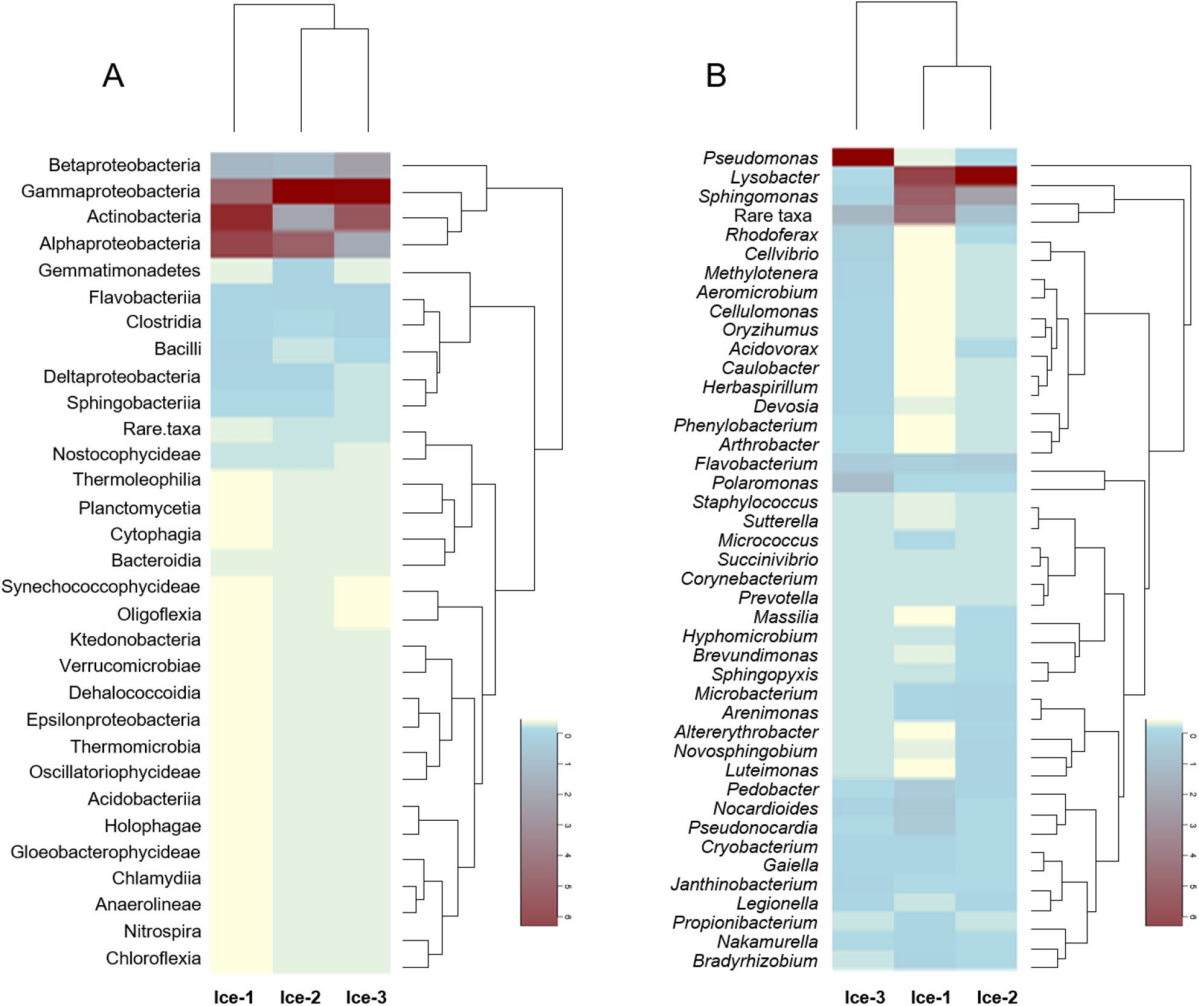
Heat-map analysis of the relative abundance of members of cave-ice prokaryotic communities at class (**A**) and genus (**B**) levels in Ice-1, Ice-2 and Ice-3. Phylotypes whose relative abundances at class level were < 0.1% and phylotypes whose relative abundances at genus level were < 0.05% were amalgamated and classified as “Rare taxa”.

Among the 31 classes detected in this study, members of Gammaproteobacteria were most abundant and represented 20.1% (Ice-1), 45.3% (Ice-2) and 42.5% (Ice-3) of total detected phylotypes (Fig. 3A). This proteobacterial group was also most abundant in the ice from Scărişoara Cave^14^. Actinobacteria represented the second most abundant group of phylotypes, with its relative abundances declining from 30.8% in Ice-1 to 26.2% in Ice-3 and 11.7% in Ice-2. Other notably abundant classes were Alpha- and Betaproteobacteria, whose abundances ranged from 9.6 to 26.3% and from 6.9 to 12.3%, respectively.

Representatives of phylum Bacteroides, classes Flavobacteria, Sphingobacteria, Bacteroidia, and Cytophagia, represented 0.08–2.0% of detected phylotypes. Firmicutes were represented by classes Clostridia (1.0–1.8%) and Bacilli (0.6–2.1%), while the abundance of Gemmatimonadetes (0.3–1.8%), the only class of the phylum, was similar to that described in soil, around 2.0%^50^, and is probably a consequence of soil deposition in the ice. The relative abundances of the remaining 17 classes were less than 1.0%. These included Cyanobacteria, which were represented by the classes Nostocophycideae, Oscillatoriophycideae and Synechococcophycidae.

At the genus level, ice cave sequences from three samples corresponded to 442 genera, but only 43 genera had abundances greater than 0.5% in all samples (Fig. 3B). For comparison, 526 genera were identified in Scărişoara Cave^14^. The most abundant genera in the Paradana samples were *Pseudomonas* and *Lysobacter* (Gammaproteobacteria); however, their abundance varied within the samples.

*Pseudomonas* spp. are known to thrive in cold environments, precipitation and clouds^51, 52^. In this study, *Pseudomonas* dominated sample Ice-3 (53.2%), yet was scarce in Ice-1 (0.5%) and Ice-2 (0.2%). In a contrast, *Lysobacter* phylotypes are usually found in waters and soils^53–56^ and were dominant in Ice-1 (23.6%) and Ice-2 (56.6%), but represented only a minor portion of phylotypes in Ice-3 (0.7%). *Lysobacter* members are pigment producers; for example, *L. oligotrophus* synthesises a water-soluble melanin pigment^57^, which could be partly responsible for dark coloration of the ice.

*Sphingomonas* spp. represented the third most abundant phylotypes. Members of the family Sphingomonadaceae were commonly found in cave drips^58^. In Scărişoara Cave, members of *Sphingomonas* were particularly common in recently formed ice strata ^14^. In Paradana the abundance of *Sphingomonas* phylotypes followed that of *Lysobacter* spp. and was 19.2% and 14.5% in Ice-1 and Ice-2, but only 1.1% in Ice-3.

It is possible that the above mentioned dominant bacteria interact with each other, but the nature of any interactions is not yet elucidated. For example, *Lysobacter* is commonly know for its antagonism towards other bacteria under nutrient-poor conditions, but in a direct assay of *Lysobacter* predation, the population of *Pseudomonas fluorescens* was not affected^59^. Furthermore, plant-colonizing *Sphingomonas* protect plants against pathogenic *P. syringae*^60, 61^ but there are reports that *Sphingomonas* can be outcompeted by fast growing *Pseudomonas* ^62^.

It is important to mention specific genera that represented a minor portion of diversity. *Polaromonas* phylotypes are particularly abundant in polar and high-elevation environments owing to the dispersal of dormant cells by air currents^63^. In cave ice, *Polaromonas* phylotypes represented a minor proportion of the diversity. They were most abundant in Ice-3 (6.9%), and scarce in Ice-1 (1.1%) and Ice-2 (0.7%). Other notable genera whose abundance exceeded 0.5% were *Flavobacterium* (abundance range 2.7–3.6%), *Nocardioides* (abundance range 0.8–3.7%), *Pseudonocardia* (0.6–3.3%) and *Pedobacter* (0.5–2.9%), which are common in a variety of natural environments. In particular *Flavobacterium* is common in a variety of cryospheric habitats^64^.

## Conclusions

Study of recently formed ice in Paradana revealed no evidence of either uniform depositional conditions or uniform microbial representation, even in the same ice stratum, indicating compartmentalization within a larger ice block. Paradana, and caves having similar geological settings and climatological conditions, are subjected to repeating cycles of freezing–melting events, and ice deposition, that influence the microbiome. In general, ice as a habitat in karst caves at lower altitudes in temperate zones is currently prone to wasting. Soluble organic compounds transported by percolation water and melted snow mitigate osmotic stress, reduce the environmental extremity and support the dynamic nature of the cave-ice microbiome. Proteobacteria, particularly from the class Gammaproteobacteria, represent the core of the cave-ice microbiome in this recently formed ice ecosystem. *Pseudomonas* and *Lysobacter* were the most abundant genera identified in Paradana. The roles and abundance of phototrophs might also be significant, particularly in caves or parts of caves with available sunlight. It remains unconfirmed, however, whether specific microorganisms or concentrations of microorganisms can provide reliable boundary markers for regular episodes of ice formation in caves.

## Materials and methods

### Site description

The studied ice cave Velika ledena jama v Paradani (Big Ice Cave in Paradana; hereafter Paradana, 45°59′17.39"N 13°50′43.89"E, Slovenian cave cadastre no. 742) is situated on the Trnovski gozd karst plateau (1130 m a.s.l.) at the foot of the Golaki ridge (Mt. Golak, 1495 m a.s.l.). Annual precipitation on the plateau averages 1700–3200 mm, with its maximum in October and November, and with snow-cover present from November until the end of April. The mean annual temperature on the plateau at 1200 m a.s.l. is around 4.5 °C.

The entrance to Paradana is at the bottom of a large, 50 m-deep doline, where snow accumulates during the winter and persists until summer due to cold-air circulation from the cave. This creates favourable conditions for temperature and vegetation-zone inversion^65^. The cave is more than 7,000 m long and 850 m deep. Because of the distinctive funnel-shaped morphology of the cave entrance, snow, soil, leaves, and tree branches slide into the cave. Permanent ice appears at a depth of about 35 m inside the cave. Most of the cave-ice forms in late winter from water that percolates through the ceiling and meltwater from the snow that accumulates in the entrance area. Water flows for some distance along the chilled passage floor before it freezes. Snowmelt produces additional water that partly inundates the passages and creates a frozen lake. This lake was used as a source of ice for cooling and food preservation until the beginning of the twentieth century. The commercial exploitation of ice was documented as early as 1867. Based on available documentation, the ice volume around 1917 was comparatively greater than currently. In the period from the 1950s to 1977 the cave entrance was completely ice-filled, and in 1978 the ice melted and further exploration of the cave became possible. During the following decade, the ice body changed its volume notably. Since 2000, the ice block has predominantly been shrinking in volume and surface area^66^. Relative mild winters in recent years accelerated the ice melt trend markedly.

**Figure 4:**
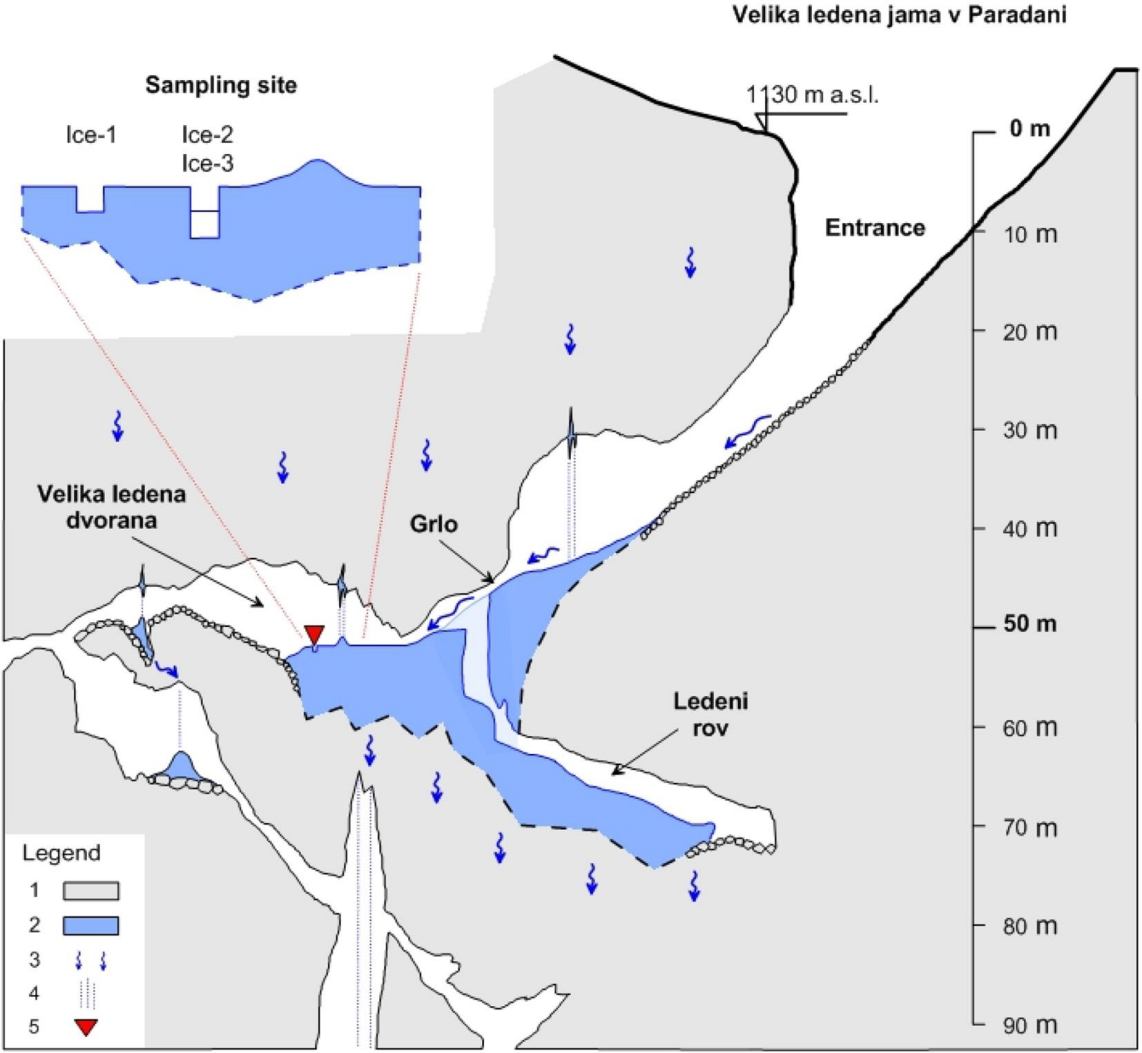
Paradana Ice Cave showing sampling site location (1-limestone, 2-ice, 3-water flow, 4-percolation water, 5-sampling site).

The floor of the Velika ledena dvorana cave chamber contains a huge ice body lying over collapsed boulders (Fig. 4). Here, the ice is at least 12 m thick, with an estimated volume of about 8,000 m^3^. The cave’s continuation beyond Velika ledena dvorana leads to smaller passages, followed by vertical pits and ramps. In this part of the cave, ice is present only to a depth of about − 200 m.

Both the formation and the preservation of ice have been attributed to the effects of air circulation^66, 67^. The temperature of the Trnovski gozd karst massif and of the air inside its caves is relatively stable of about 4–5 °C. In winter, when the external temperature falls below 4 °C, an airflow is created leading to transfer of cold air from the surface into the cave. This air re-emerges from the karst massif at higher altitude (about 1400 m a.s.l.) where numerous patches of melting snow-have been observed. Initially the snowmelt is due to the effect of displaced air. The cold air flows through a system of interconnected cavities within the Golaki ridge. The height difference of about 270 m is great enough to create a “chimney effect”^68^. In the summer, the air temperature at the surface rises above the temperature within the cave system and the airflow reverses its direction. It moves from the lower parts of Paradana, passes the ice body, is cooled to about 1 °C, and exits the cave. Both winter and summer temperature regimes and flow directions are stable. During the summer months, cold airflow creates and maintains a large, stable, volume of cold air in the doline that holds the cave entrance.

### Sampling

The exact micro-locations for ice sampling were selected specifically, away from the margins of the large ice body, to ensure comparable ice strata in the samples and to avoid material such as branches, stones and other detritus that are scattered in the ice block. Two ice blocks, Ice-1 and Ice-2–Ice-3, approximately 1.5 m apart, were cut with a chain saw from the frozen lake in Velika ledena dvorana on 09 May 2016 (Fig. 4). At the sampling site there was very weak indirect natural illumination (< 1 lx, < 0.01 µmol photons/m^2^ s, < 0.1 W/m^2^, LI-1500, LI-COR). Ice-2 was cut aseptically from the ice block Ice-2–Ice-3 to obtain the same ice stratum (approximately 15 cm thick) as in Ice-1. Compared to Ice-1 and Ice-2, the Ice-3 block was less transparent, with more dark inclusions of particulate organic material, and it was formed before the Ice1–Ice2 stratum.

Monthly composite precipitation samples were collected in Postojna between August 2015 and October 2017 (45°46′31.55"N 14°12′47.64"E, 553 m a.s.l., about 38 km from Paradana) and analysed for stable isotopes in order to construct the local meteoric water line (LMWL). Additionally, mean daily air temperature and daily precipitation were measured at Postojna. LMWLs calculated for other precipitation stations in Slovenia^27, 69^ were also examined.

### Physicochemical analyses

Contact surfaces of the ice blocks were sterilized using a gas burner before melting in the laboratory. Complete ice melt occurred after 48 h of incubation at room temperature, with the blocks yielding 3.5–5.0 litres of water. All ice samples included layers containing dark inclusions of organic matter, which affected the transparency of both the ice and the water after melting. The water was sub-divided aseptically to provide aliquots for subsequent analyses: 500 ml was used for chemical analyses, 5 ml for cultivation of microbes, total ATP concentration and flow cytometry, 5 ml for isotopic analyses, and 1.5 litre of sample water was used to extract each total community DNA. DNA was isolated from filters after membrane filtration of water through 0.22 µm pore-size filters.

Alkalinity (SM 2320), hardness (SM 2340), and concentrations of chloride (SM 4500-Cl^−^), nitrate (SM 4500-NO_3_^−^), ortho-phosphate (SM 4500-P), sulphate (SM 4500-SO_4_^2−^), and total organic carbon (SM 5310 TOC) were determined following standard methods^70^. Electrical conductivity (EC) and pH were measured using a portable WTW MultiLine P4 meter.

Stable isotopes of oxygen and hydrogen were measured by Picarro 2120i and 2130i water isotope analysers using previously described protocols^71, 72^. The samples were analysed in triplicates and seven injections were used per sample. The values were expressed in the conventional δ notation (‰) with respect to Vienna Standard Mean Ocean Water. The analytical accuracy was better than 0.2‰ for δ^18^O and better than 1.0‰, for δ^2^H. The modified intersection method was used to obtain the isotopic composition of the precipitation that formed the cave ice^73^. Correlation of δ^2^H and deuterium excess d (d = δ^2^H–8δ^18^O) in water from ice was first checked to exclude kinetic fractionation during water freezing. Then, intersection of the freezing line determined by stable isotopes in samples Ice-1 to Ice-3 and the LMWL constructed for precipitation at Postojna provided the probable isotopic composition of the water that formed the sampled cave ice.

### Concentration of microorganisms and isolation of phototrophs

Microbial load in the samples was measured as total adenosine triphosphate (ATP) (AquaSnapTotal, Hygiena, USA), and expressed in Relative Light Units (RLU). Total cell counts and cell viability were determined by flow cytometry. The Cell Viability Kit with Liquid Counting Beads (BD Biosciences) was used according to the manufacturer's instructions on a FACS Canto Flow Cytometer (Becton Dickinson) equipped with three lasers. At least 30,000 events were acquired. Data analysis was carried out using BD FACSDiva 6.1.2 software (Becton Dickinson)^74^.

Colony counts in water samples were expressed as colony-forming units (CFUs) per ml using the following media: RIDACOUNT Total Aerobic Count (R-Biopharm); RIDACOUNT Yeast&Mold Rapid (R-Biopharm); nutrient agar (NA, Fluka); R2A agar (Merck); malt extract agar (MEA, Fluka); tap water agar (TWA) and percolation water agar (PWA). The PWA contained cave percolation water from Planina Cave (Planinska jama, 45°49′12.68"N 14°14′44.31"E, Slovenian cave cadastre no. 748) with the following physicochemical characteristics: pH 8.27; EC 525 μS/cm; chloride 1.3 mg/l; nitrate 15.7 mg/l; sulphate 4.3 mg/l; ortho-phosphate 0.0 mg/l; hardness 261 CaCO_3_ mg/l; and alkalinity 256 CaCO_3_ mg/l. All plates were incubated under aerobic conditions. In addition, a subset of RIDACOUNT plates were incubated anaerobically using GENbag anaerobic airtight bags (Biomérieux). Plates were cultivated at 37 °C for 2 days, 20 °C for up to 14 days, 10 °C for 28 days, and 5 °C for 28 days.

To estimate bacterial r/K growth strategy, water samples were inoculated onto R2A agar plates and incubated at 20 °C. Microbes that grew after 3 days were classified as r-strategists (fast growing opportunistic species at low population densities), and microbes that grew within an additional 4–7 days as K-strategists^75^.

To isolate phototrophs, one ml of water was inoculated in 50 ml of BG11 liquid medium (Promega) and incubated in a laboratory with moderate natural illumination and at room temperature. After 33 weeks, algae were identified by examination under a Nikon Eclipse TE 300 phase-contrast microscope equipped with a digital camera and Nikon software NIS Elements. Cells were identified according to the specialized key of Kol and Flint^34^. For cell-size estimation, 30 randomly selected cells were measured, and the average size and standard deviation were calculated (Supplementary Fig. S3).

### Total community DNA extraction, 16S amplicon sequencing, and bioinformatic analysis

Water (1.5 l) from melted ice blocks was filtered through two 0.22 μm pore-size filters (47 mm in diameter, Millipore). Filters were used to isolate total community DNA using a MoBio PowerWater DNA Isolation kit. DNA purity and concentration were determined spectrophotometrically using standard absorbance at 260 nm and 280 nm on a Perkin Elmer Lambda 25 UV–VIS Spectrometer equipped with UV CellTray (Hellma Analytics). The 260/280 absorbance ratios ranged from 1.63 to 1.74. The best DNA results from the duplicates, in terms of quantity and purity (A_260_/A_280_), were used for subsequent sequencing.

Amplification of 16S rDNA, library preparation and IonTorrent sequencing were performed at Omega, Ljubljana (Slovenia). Each sample was sequenced once. Ion 16S Metagenomics Kit (ThermoFisher Scientific, Waltham, MA, USA) was used to amplify V2-4–8 and V3-6, 7–9 regions of 16S rRNA in two separate PCR reactions consisting of initial denaturation at 95 °C for 10 min, 25 cycles of denaturation at 95 °C for 30 s, annealing at 58 °C for 20 s, and polymerisation at 72 °C for 20 s, followed by a final extension step at 72 °C for 7 min.

Amplified DNA was purified using Agencourt AMPure XP (Beckman Coulter, Brea, CA, USA). Purified PCR products were quantified on LabChip GX Touch using HT DNA High Sensitivity Reagent Kit (both PerkinElmer, Waltham, MA, USA). Fifty nanograms of amplicons were processed to make the DNA library using Ion Plus Fragment Library Kit and barcode adapters Ion Xpress Barcode Adapters Kit (both ThermoFisher Scientific, Waltham, MA, USA). Size distribution and number of processed libraries were evaluated with the LabChip GX instrument (PerkinElmer, Waltham, MA, USA). Each sample was adjusted to 20 picomolar concentration. Equal volumes of all samples were combined and processed in emulsion PCR and enrichment steps with One-Touch 2 and One-Touch ES systems using the Ion PGM Hi-QT View OT2 Kit (all ThermoFisher Scientific, Waltham, MA, USA). Finally, the libraries were sequenced in a 316 v2 chip on the Ion Personal Genome Machine (PGM) using the Ion PGM Hi-QT View Sequencing Kit following the manufacturer’s protocol (all ThermoFisher Scientific Waltham, MA, USA).

Raw reads were analysed using cloud service Ion Reporter Software v.5.10, which implements QIIME's open-source bioinformatics pipeline^76^. Primer sequences were removed from the reads, and reads shorter than 150 bp were removed from the dataset. The sequences were aligned against the Curated Greengenes 16S reference library v13.5. Minimum alignment coverage was set to 90% and the read abundance filter set to 10 reads. Genus-level OTUs were determined at 97% sequence identity and species-level OTUs at 99% sequence identity. Rarefaction curves were determined based on Chao1 metrics. Vegan Community Ecology^77^ and Bioconductor^78^ packages were used within an R free-software environment^79^.

to calculate the alpha-diversity measures Chao1, ACE and Shannon index, to produce heatmaps, and to generate a Venn diagram.

## Data availability:

raw sequencing reads were deposited in the NCBI Sequence Read Archive under the BioProject accession number PRJNA635015 corresponding to BioSample accession numbers of SAMN15015198 (sample Ice-1), SAMN15015199 (sample Ice-2) and SAMN15015200 (sample Ice-3).

## Supplementary Information

Supplementary Information
