# Supplementary material for: Microbiota entrapped in recently-formed ice: Paradana Ice Cave, Slovenia
Source: Sci Rep. 2021 Jan 21;11:1993. doi: 10.1038/s41598-021-81528-6 (PMC7820503; doi:10.1038/s41598-021-81528-6)
Supplement: Supplementary file 1 — Supplementary Information [file 41598_2021_81528_MOESM1_ESM.docx]

**Microbiota entrapped in recently-formed ice:** **Paradana Ice Cave, Slovenia**

Janez Mulec^1*^, Andreea Oarga-Mulec^2,3^, Ladislav Holko^4^, Lejla Pašić^5^, Andreja Nataša Kopitar^6^, Tina Eleršek^7^, Andrej Mihevc^1^

**Supplementary Information**


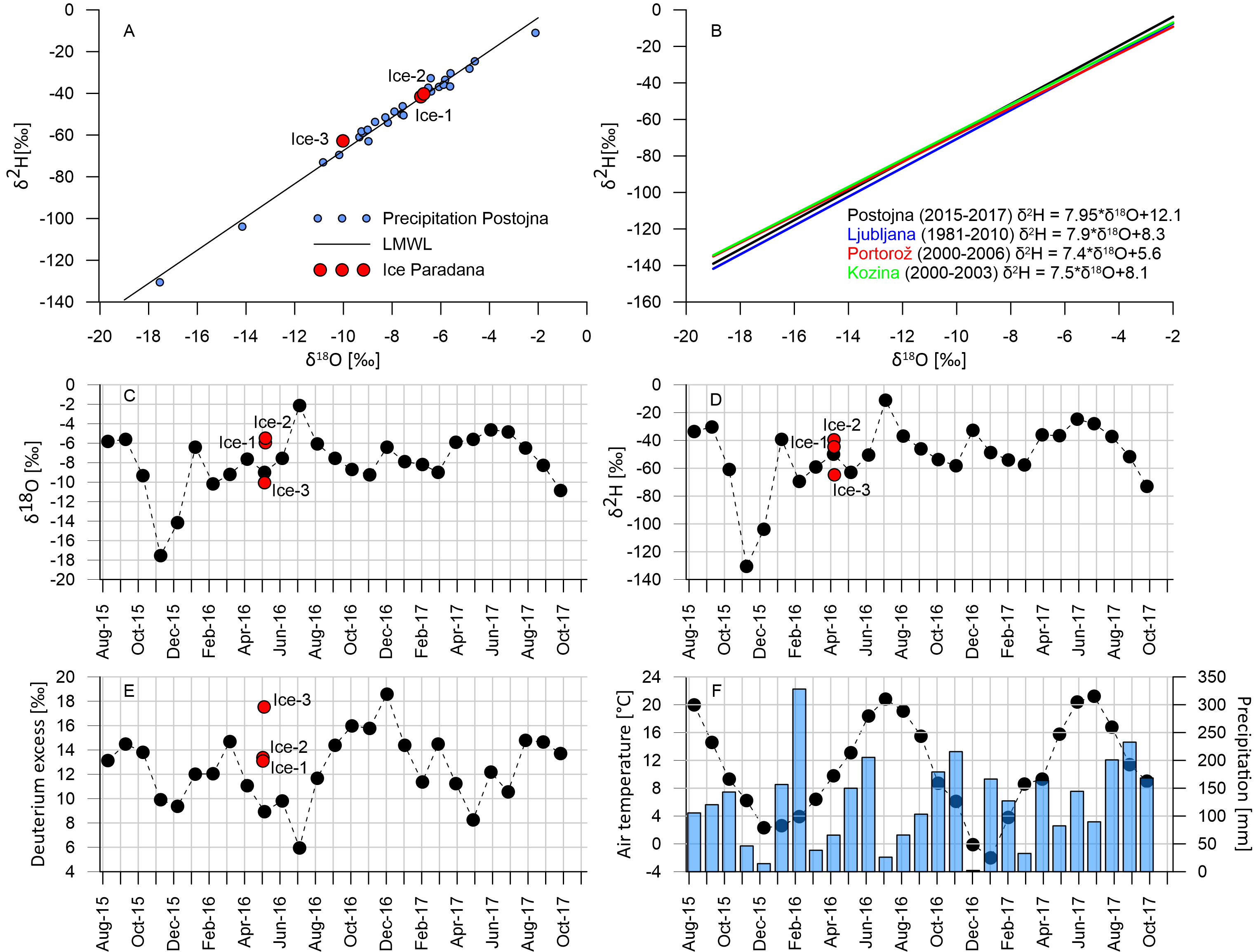


Figure S1: Isotopic analyses. A: Isotopic composition of monthly precipitation water at Postojna between August 2015 and October 2017, derived local meteoric water line (LMWL) and position of ice samples from Paradana Ice Cave. B: LMWLs calculated for other precipitation stations in Slovenia (data from IAEA/WMO, 2019). C–E: temporal variability of the isotopic composition of monthly precipitation at Postojna; the red dots represent values for ice from Paradana Ice Cave. F: monthly mean air temperature and precipitation totals at Postojna between August 2015 and October 2017.


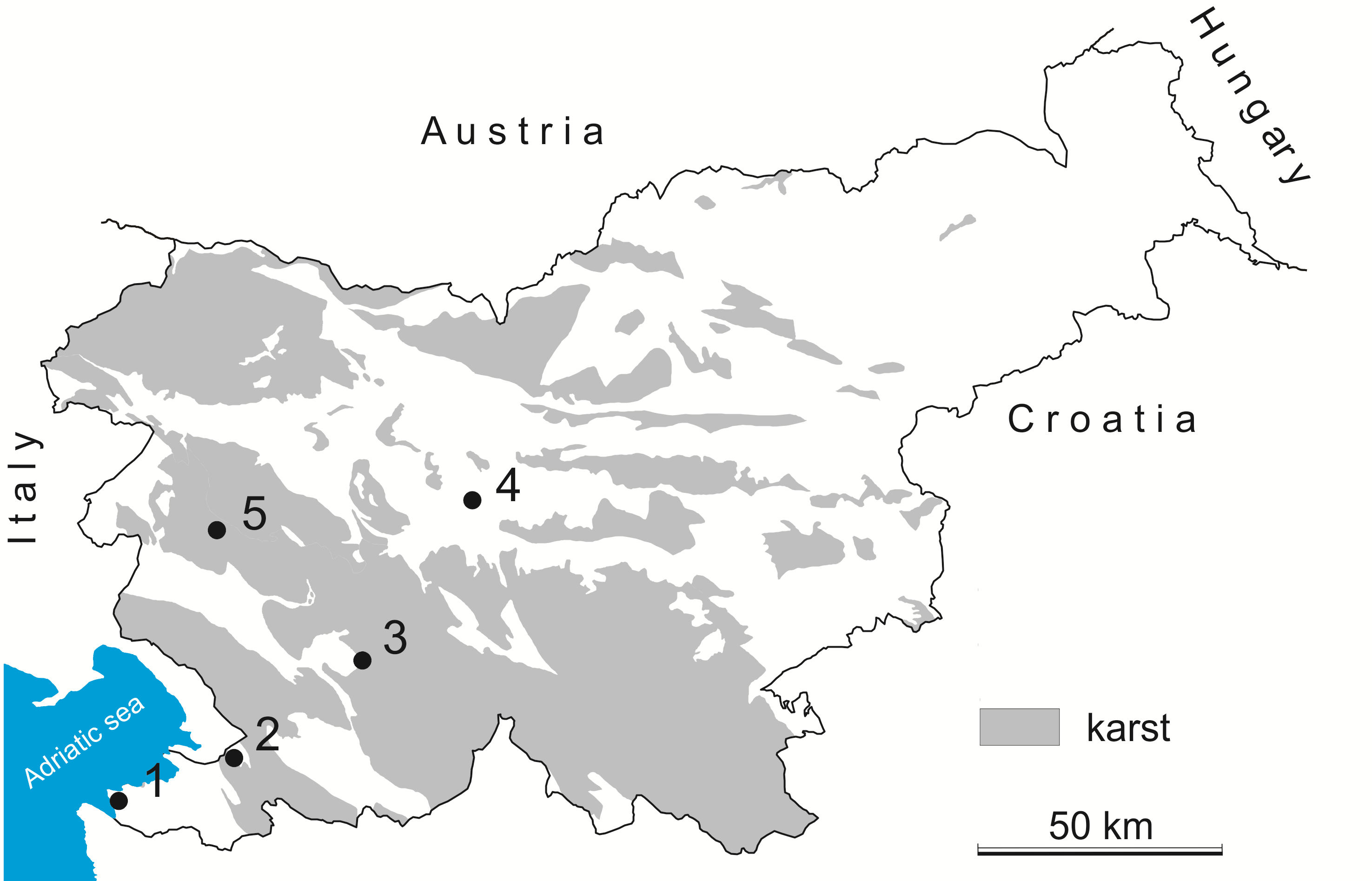


Figure S2: Locations of precipitation stations (1 – Portorož; 2 – Kozina; 3 – Postojna; 4 – Ljubljana) and Paradana Ice Cave (5) in Slovenia. Map was created using Surfer 14 (Golden Software, https://www.goldensoftware.com/)


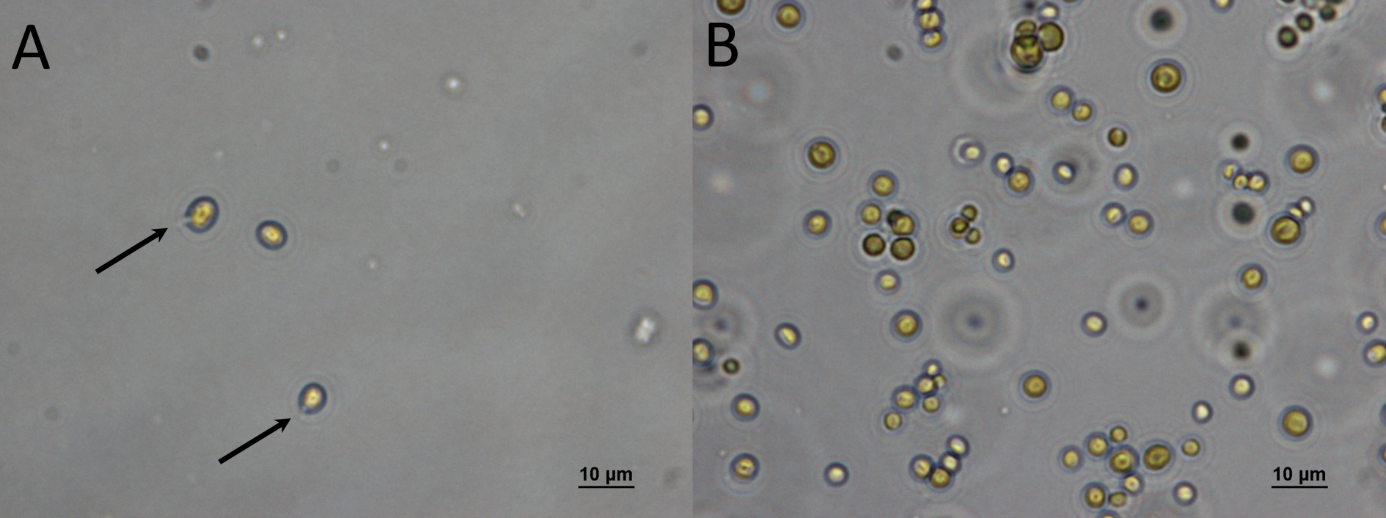


Figure S3: Cultures of algae from Paradana Ice Cave viewed under a phase-contrast microscope. A: Cells of *Ellipsoidion perminimum* Pascher var. *cryophila* are typically spherical or ovoid, 4–6 µm long and 1–4 µm wide, with one chloroplast. Measurements revealed cells 3.8 ± 0.6 µm long and 2.4 ± 0.6 µm wide (n=30). Drops of red oil are present; pyrenoids are absent. The alga reproduced by forming 2–4 autospores, which were liberated by gelatinization of the mother-cell wall. Other stages were not recognized. The variety is distinguished by the size of the chloroplast and by its habitat^34^. Arrows indicate flagella. B: Cells of *Chloridella glacialis* Kol sp. nov. are spherical, 0.5–1.5 µm in diameter, and contain one chloroplast without a pyrenoid. The measurements revealed spherical cells with a diameter of 2.0 ± 0.6 µm (n=30). Reproduction is restricted to the formation of 2–4–(16) autospores, which are liberated by gelatinization of the mother-cell wall. This species differs from *C. simplex* Pascher both in size and in habitat^34^.


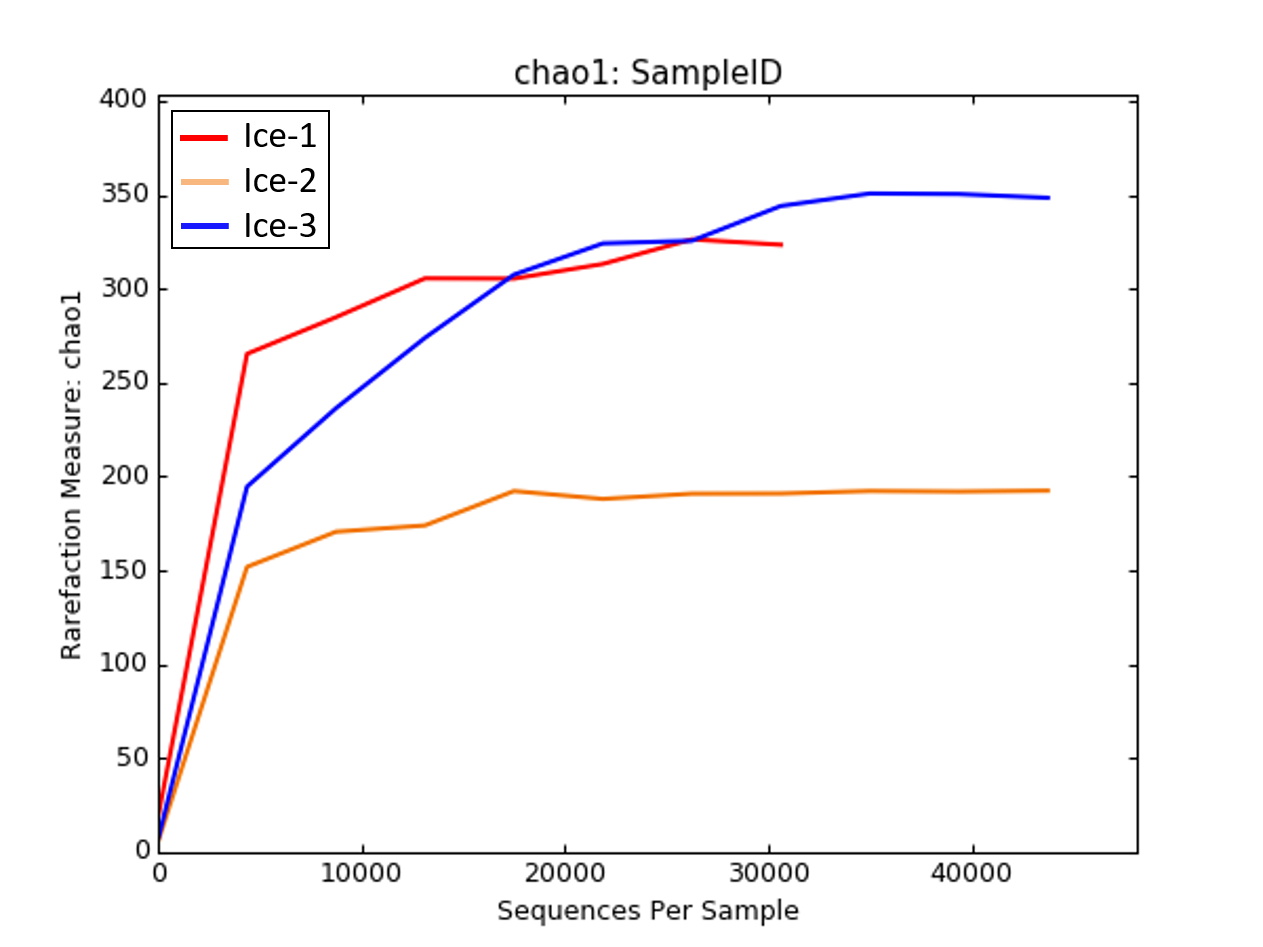


Figure S4. Rarefaction curves based on Chao1 metrics for samples Ice-1, Ice-2 and Ice-3 from Paradana Ice Cave. R free-software (version 3.6.1) was used to create rarefaction curves (www.r-project.org).


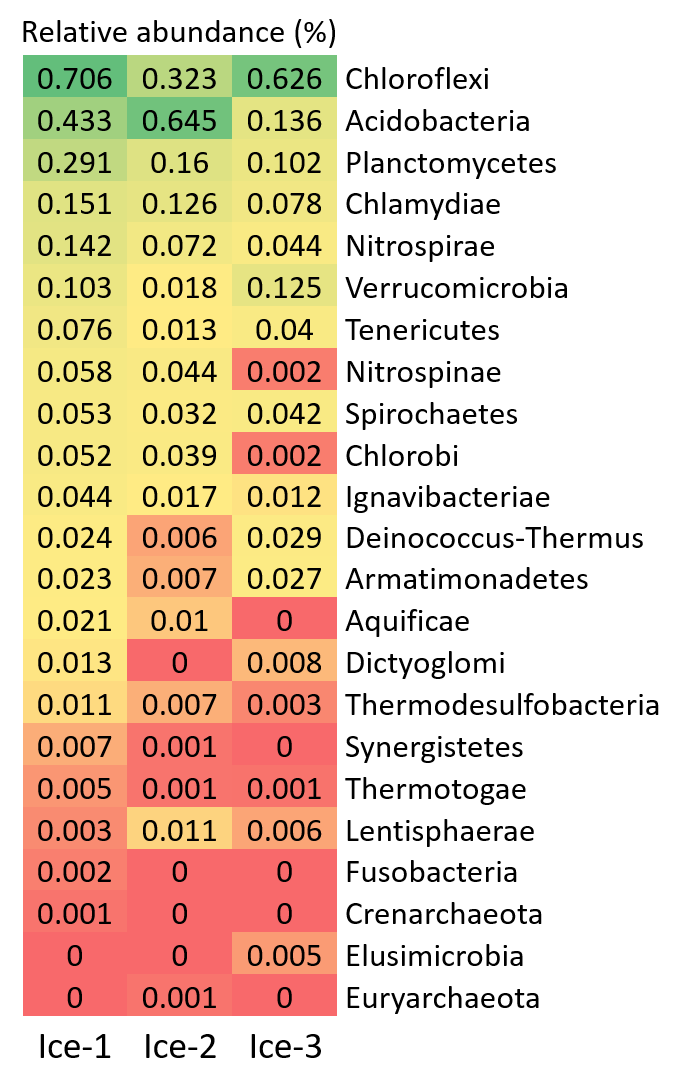


Figure S5. Relative abundance of phyla that represented < 1.0% of detected phylotypes (“rare phyla”) in the Paradana Cave ice samples.
